# Comparison of the TIMI, GRACE, PAMI and CADILLAC risk scores for prediction of long-term cardiovascular outcomes in Taiwanese diabetic patients with ST-segment elevation myocardial infarction: From the registry of the Taiwan Society of Cardiology

**DOI:** 10.1371/journal.pone.0229186

**Published:** 2020-02-13

**Authors:** Yung-Ta Kao, Yi-Chen Hsieh, Chien-Yi Hsu, Chun-Yao Huang, Ming-Hsiung Hsieh, Yung-Kuo Lin, Jong-Shiuan Yeh

**Affiliations:** 1 Division of Cardiology, Department of Internal Medicine, Taipei Medical University Hospital, Taipei, Taiwan; 2 Cardiovascular Research Center, Taipei Medical University Hospital, Taipei, Taiwan; 3 Taipei Heart Institute, Taipei Medical University, Taipei, Taiwan; 4 Division of Cardiology, Department of Internal Medicine, School of Medicine, College of Medicine, Taipei Medical University, Taipei, Taiwan; 5 Professional Master Program in Artificial Intelligence in Medicine, College of Medicine, Taipei Medical University, Taipei, Taiwan; 6 PhD Program of Neural Regenerative Medicine, College of Medical Science and Technology, Taipei Medical University, Taipei, Taiwan; 7 PhD Program in Biotechnology Research and Development, College of Pharmacy, Taipei Medical University, Taipei, Taiwan; 8 Master Program in Applied Molecular Epidemiology, College of Public Health, Taipei Medical University, Taipei, Taiwan; 9 Division of Cardiovascular Medicine, Department of Internal Medicine, Taipei Municipal Wan-Fang Hospital, Taipei, Taiwan; Osaka University Graduate School of Medicine, JAPAN

## Abstract

Acute coronary syndrome (ACS) patients with diabetes have significantly worse cardiovascular outcomes than those without diabetes. This study aimed to compare the performance of The Thrombolysis In Myocardial Infarction (TIMI), Global Registry of Acute Coronary Events (GRACE), Primary Angioplasty in Myocardial Infarction (PAMI), and Controlled Abciximab and Device Investigation to Lower Late Angioplasty Complications (CADILLAC) risk scores in predicting long-term cardiovascular outcomes in diabetic patients with ST-segment elevation myocardial infarction (STEMI). From the Acute Coronary Syndrome-Diabetes Mellitus Registry of the Taiwan Society of Cardiology, patients with STEMI were included. The TIMI, GRACE, PAMI, and CADILLAC risk scores were calculated. The discriminative potential of risk scores was analyzed using the area under the receiver-operating characteristics curve (AUC). In the 455 patients included, all four risk score systems demonstrated predictive accuracy for 6-, 12- and 24-month mortality with AUC values of 0.67–0.82. The CADILLAC score had the best discriminative accuracy, with an AUC of 0.8207 (p<0.0001), 0.8210 (p<0.0001), and 0.8192 (p<0.0001) for 6-, 12-, and 24-month mortality, respectively. It also had the best predictive value for bleeding and acute renal failure, with an AUC of 0.7919 (p<0.05) and 0.9764 (p<0.0001), respectively. Patients with CADILLAC risk scores >8 had poorer 2-year survival than those with lower scores (log-rank p<0.0001). In conclusion, the CADILLAC risk score is more effective than other risk scores in predicting 6-month, 1-year, and 2-year all-cause mortality in diabetic patients with STEMI. It also had the best predictive value for in-hospital bleeding and acute renal failure.

## Introduction

Diabetes mellitus (DM) is associated with poor outcomes in patients with coronary artery disease (CAD) [1]. In Taiwan, acute coronary syndrome (ACS) patients with diabetes had significantly worse outcomes than those without DM, including all-cause death and combined results for death, re-infarction, and stroke [2]. To improve ACS-related mortality and morbidity in Taiwan, the Acute Coronary Syndrome-Diabetes Mellitus Registry of the Taiwan Society of Cardiology (TSOC ACS-DM Registry) was established to assess the quality of care for ACS patients with DM. This study was conducted to determine accurate risk stratification in the management of ACS patients with DM. Several risk scores have been developed in the last 20 years to stratify patients hospitalized with ACS [3–8]. The most widely used risk score is the Thrombolysis In Myocardial Infarction (TIMI) algorithm, which is simple to calculate and is derived from selected clinical-trial cohorts. For ST-segment elevation myocardial infarction (STEMI) patients, the TIMI score is based on eight clinical indicators available upon admission, with scores ranging from 0 to 14. The second most used score is the Global Registry of Acute Coronary Events (GRACE) risk model, which uses eight variables and is applicable to the entire spectrum of ACS. The Primary Angioplasty in Myocardial Infarction (PAMI) score is based on clinical and electrocardiographic characteristics. The PAMI risk score, with a range of 0 to 15 points, was found to be a strong predictor of late mortality in STEMI patient undergoing primary percutaneous coronary intervention (PCI) [8]. Finally, the Controlled Abciximab and Device Investigation to Lower Late Angioplasty Complications (CADILLAC) risk score incorporates the measurement of baseline left ventricular (LV) function. It is the single most powerful predictor of survival in ACS patients [3]. For patients with STEMI undergoing PCI, TIMI, PAMI, or CADILLAC risk scores all provide important prognostic information and enable accurate identification of high-risk patients [4]. Furthermore, TIMI and GRACE risk scores predict 5-year all-cause mortality well in patients with STEMI treated with primary PCI [5]. Table 1 features the components of these risk-scoring models. However, these risk scores were developed by enrolling patients mostly from Western countries. Although these risk scores have been externally validated in the general population for predicting all-cause death and re-myocardial infarction from the short term to a 1-year follow-up period, there is limited data on the ability of these risk score systems to predict long-term cardiovascular events and in-hospital outcomes, including acute renal failure or bleeding, in specific populations such as DM patients. The aims of this present prospective observational study were to compare the prognostic value of four risk scores in the risk stratification of Taiwanese diabetic patients with STEMI, and to examine whether these risk scores could be applied to predict either short-term in-hospital outcomes or future cardiovascular events up to two years after STEMI.

**Table 1:** Risk scoring models and their components.

Model components	TIMI for STEMI	PAMI	CADILLAC	GRACE
**Age**	+	+	+	+
**Low blood pressure**	+			+
**Heart rate**	+	+		+
**Killip class**	+	+	+	+
**Diabetes mellitus**	+	+		
**Hypertension**	+			
**Angina pectoris**	+			
**Anterior MI or LBBB**	+	+		
**Weight**	+			
**Ischemia time**	+			
**TIMI flow**			+	
**Ejection fraction**			+	
**Anemia**			+	
**Three-vessel disease**			+	
**ST-segment deviation**				+
**Creatinine/renal insufficiency**			+	+
**Cardiac arrest**				+
**Increased cardiac markers**				+

**Abbreviations:** CADILLAC, Controlled Abciximab and Device Investigation to Lower Late Angioplasty Complications; GRACE, Global Registry of Acute Coronary Events; LBBB, left bundle branch block; MI, myocardial infarction; PAMI, Primary Angioplasty in Myocardial Infarction; STEMI, ST-segment elevation myocardial infarction; TIMI, Thrombolysis In Myocardial Infarction.

## Methods

The study complied with the Declaration of Helsinki and was approved by the Taipei Medical University-Joint Institutional Review Board (Ethics Reference: 201312017). Written informed consent was obtained from all study participants. All patients were participants in the TSOC ACS-DM Registry. This is a prospective, nationwide, multicenter, non-interventional, observational clinical registry–based study, the detailed recruitment procedures of which have been published [9]. In brief, the inclusion criteria included patients 1) who were admitted to the hospital with ACS within the previous 30 days; 2) with a history of type 2 DM or newly-diagnosed DM defined according to World Health Organization (WHO) criteria; 3) aged ≥20 years; and 4) who agreed to provide informed consent. The exclusion criteria included ACS accompanied with or precipitated by significant comorbidity such as severe gastrointestinal bleeding, trauma, peri-operative or peri-procedural myocardial infarction (MI), or participation in an investigational drug trial. In total, 1,534 ACS patients with DM, including 455 STEMI patients, 750 non-ST-segment elevation myocardial infarction (NSTEMI) patients, and 329 unstable angina (UA) patients, were registered between January 2013 and December 2015. Approval for use of the TSOC ACS-DM Registry was acquired from the Institutional Review Board of each participating hospital. All subjects completed signed informed consent and permission to record follow-up outcomes.

Patients with type 2 DM were diagnosed according to the criteria of the American Diabetes Association and the WHO. Those who had already taken oral hypoglycemic agent(s), had hemoglobin A1C levels of 6.5% or higher, or fasting plasma glucose 126 mg/dL or higher, or 2-hour post-prandial blood sugar 200mg/dL or higher, were considered to be DM patients [10].

ACS refers to a spectrum of conditions compatible with acute myocardial ischemia and/or infarction that are usually due to an abrupt reduction in coronary blood flow [11]. Patients with ACS and elevated cardiac biomarker values are diagnosed with MI. For the sake of immediate treatment strategies such as reperfusion therapy, it is usual practice to designate MI in patients with chest discomfort or other ischemic symptoms who develop ST elevation in two contiguous leads as STEMI [12].

All data, including demographic characteristics, medical therapy, laboratory tests, and invasive measurement, including quantitative coronary analysis and TIMI flow grade assessment after PCI, were collected by physicians and study nurses. Medications or treatments upon admission, duration of hospitalization, and status at discharge were also collected. All data were submitted electronically to a central laboratory and audited for quality assurance.

Echocardiographic assessment was carried out 3–5 days after MI onset. Left ventricular ejection fraction was estimated primarily using the biplane Simpson’s formula with apical two- and four-chamber views.

The primary endpoint of interest was all-cause mortality at 6 months, 12 months, and 24 months. The secondary endpoints included in-hospital recurrent non-fatal MI, TIMI major/minor bleeding [13], new-onset cardiogenic shock, and acute renal failure. Acute renal failure is defined as a three-fold increase of serum creatinine or decrease in glomerular filtration rate of >75% or a urine output of <0.3 mL/kg per hour for >24 hours or anuria for >12 hours [14]. All records were collected from medical records by well-trained study nurses.

Numerical data are presented as the mean ± the standard deviation (SD) or median with interquartile range while categorical variables are shown as frequency with percentage. The discriminative potential of risk scores was performed using the area under the receiver-operating characteristics curve (AUC) [15]. Statistically significant differences between AUCs were examined using DeLong’s test. Calibration was evaluated with Hosmer-Lemeshow goodness-of-fit X^2^ estimates using deciles [16]. The optimal cut-off thresholds were determined by using the highest Youden index. The 2-year survival probability of each risk scores were estimated using the Kaplan-Meier method and examined by log-rank tests. All analyses were performed using SAS software version 9.4 (SAS Institute Inc., Cary, NC, USA) and STATA software version 15.0 (STATA Corp LP, College Station, TX, USA).

## Results

Table 2 reveals the baseline characteristics and medical therapy upon hospital admission of the 455 STEMI patients studied. Among these, the average age was 61.5±11.9 years and 78% were male. More than 70% of these STEMI patients had a history of hypertension. About 15% were newly diagnosed with DM during this course of hospitalization. More than half of the STEMI patients had Killip class I severity (57.4%). Upon hospital admission, 28.8% of these STEMI patients used insulin. Results of the remaining laboratory tests, including creatine kinase, glycated hemoglobin, lipid level, and invasive procedures, are presented in Table 3. More than half (53.8%) of patients had TIMI flow 0 upon hospital admission. Selective coronary angiography showed three-vessel disease in 145 (31.9%) patients.

**Table 2:** Baseline characteristics of patients and medical therapy upon hospital admission.

Characteristics	STEMI (N = 455) Mean(SD) or N(%)
**Age (years)**	61.5(11.9)
**Gender (female)**	100(22.0%)
**Height (cm)**	164.3(8.0)
**Weight (kg)**	70.1(13.4)
**Body mass index (kg/m**^**2**^**)**	25.9(3.9)
**Systolic blood pressure (mmHg)**	134.8(31.9)
**Diastolic blood pressure (mmHg)**	80.9(20.6)
**Heart rate (min**^**-1**^**)**	84.3(23.2)
**Smoker**	185(41.6%)
**History of dyslipidemia**	195(42.9%)
**History of hypertension**	321(70.6%)
**History of diabetes**	387(85.1%)
**Known CAD**	97(21.3%)
**Previous myocardial infarction**	49(10.8%)
**Previous PCI**	60(13.2%)
**Previous CABG**	7(1.5%)
**History of atrial fibrillation**	6(1.3%)
**Previous heart failure**	8(1.8%)
**COPD**	9(2.0%)
**Obstructive sleep apnea**	4(0.9%)
**Peripheral arterial disease**	9(2.0%)
**Cerebrovascular disease**	40(8.8%)
**Killip class 1**	257(57.4%)
**Killip class 2**	93(20.8%)
**Killip class 3**	43(9.6%)
**Killip class 4**	55(12.3%)
**Aspirin use**	437(96.0%)
**ACE inhibitor use**	230(50.6%)
**Angiotensin II receptor blocker use**	120(26.4%)
**Beta-blocker use**	331(72.8%)
**Statin use**	386(84.8%)
**Digoxin use**	6(1.3%)
**Diuretic use**	130(28.6%)
**IV inotropic agent use**	47(10.3%)
**Insulin use**	131(28.8%)
**Sulfonylurea agent use**	153(33.6%)
**Metformin use**	263(57.8%)
**DPP4 inhibitor use**	160(35.2%)
**Antiarrhythmic drug use**	35(7.7%)
**H2 blocker or PPI use**	166(36.5%)

**Abbreviations:** ACE, angiotensin-converting enzyme; CABG, coronary artery bypass grafting; CAD, coronary artery disease; COPD, chronic obstructive pulmonary disease; DPP4, dipeptidyl peptidase-4; H2, histamine 2; IV, intravenous; PCI, percutaneous coronary intervention; PPI, proton pump inhibitor; SD, standard deviation; STEMI, ST-segment elevation myocardial infarction.

**Table 3:** Characteristics of laboratory tests and invasive procedures.

Test/Procedures	STEMI (N = 455) Mean(SD) or N(%)
**Initial CK (U/L)**	206(105–568)
**Initial CK-MB (U/L)**	19(4–43)
**Initial troponin (ng/mL)**	0.75(0.05–12.4)
**Peak CK (U/L)**	1357(496–2728)
**Peak CK-MB (U/L)**	108(33–228.5)
**Peak troponin (ng/mL)**	25(2.76–80)
**Creatinine (mg/dL)**	1.03(0.8–1.4)
**Hemoglobin (g/dL)**	14.2(6.2)
**HbA1c (%)**	8.4(1.9)
**Total cholesterol (mg/dL)**	169.5(44.9)
**HDL (mg/dL)**	38.0(9.3)
**LDL (mg/dL)**	107.9(40.2)
**Triglyceride (mg/dL)**	152.7(117.0)
**Stenosis of culprit lesion (%)**	94.8(11.0)
**Infarct-related artery**	
**LM**	4(0.9)
**LAD**	207(46.4)
**LCx**	54(12.1)
**RCA**	179(40.1)
**Unknown**	2(0.5)
**Initial TIMI flow 0**	245(53.8)
**Number of diseased vessels**	
**0**	0(0)
**1**	188(41.3)
**2**	113(24.8)
****≥**3**	145(31.9)
**Missing**	9(2.0)

**Abbreviations:** CK, creatine kinase; CK-MB, creatine kinase myocardial band; HbA1c, hemoglobin A1c; HDL, high-density lipoprotein; LAD, left anterior descending artery; LCx, left circumflex artery; LDL, low-density lipoprotein; LM, left main; RCA, right coronary artery; SD, standard deviation; STEMI, ST-segment elevation myocardial infarction; TIMI, Thrombolysis In Myocardial Infarction.

**Fig 1:**
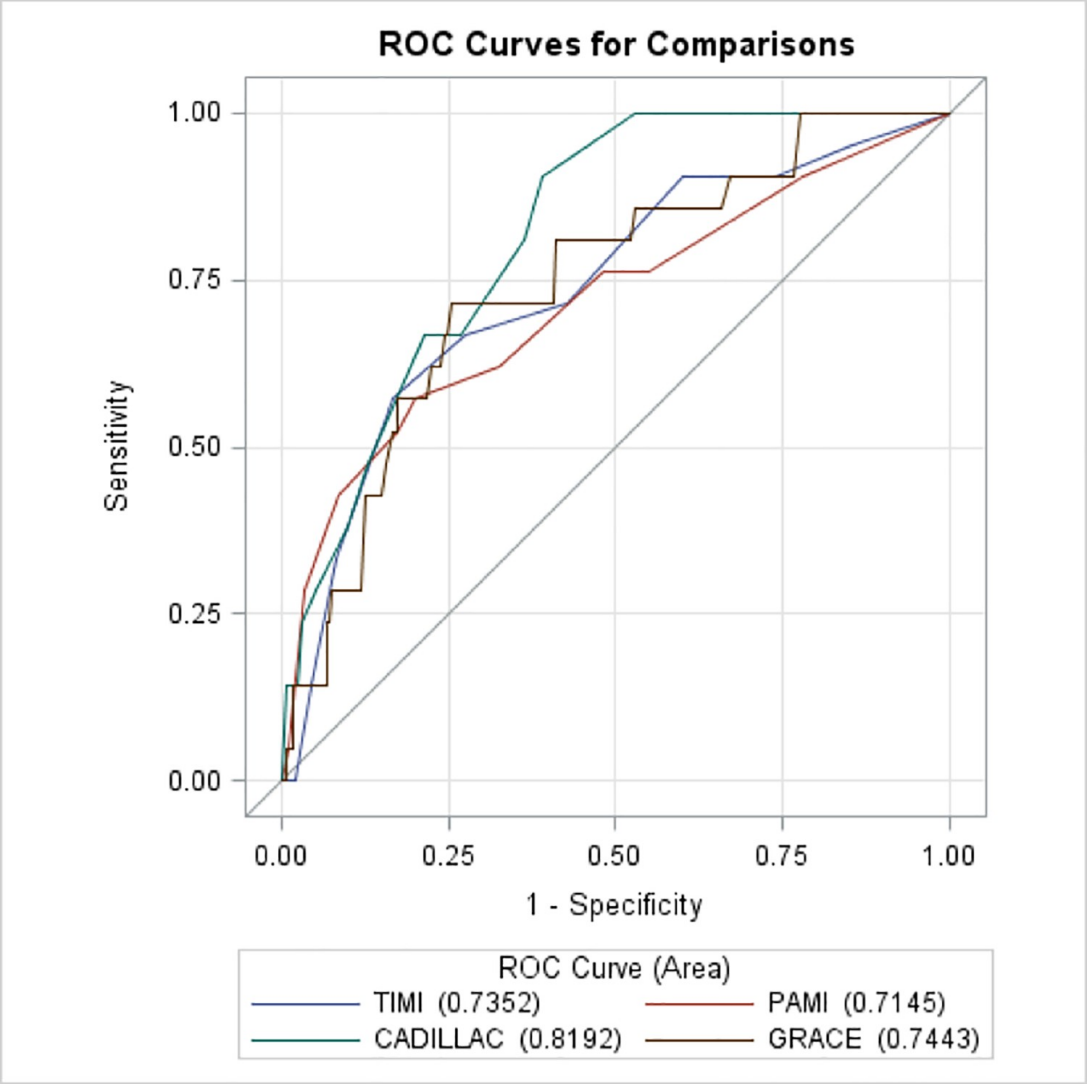
Predictive accuracy of PAMI, TIMI, CADILLAC and GRACE scoring models for 2-year mortality. According to the tertiles of each risk score, patients with the higher tertile (T3) of each risk score had unfavorable 2-year survival than those with middle tertile (T2) and lower tertile (T1) of each risk score (Fig 2). However, there was no death subjects in T1 and T2 of the CADILLAC score group. We further found the best cut-off point for the CADILLAC risk score (8 points) by using Youden’s index (Table 5). Since the CADILLAC risk score had the best predictive accuracy for mortality, we used it to estimate the survival rate at 2 years, as shown in Fig 3. Patients with CADILLAC risk scores >8 had poorer 2-year survival than those with risk scores ≤8 (both log-rank p<0.0001).

The AUC of each risk score for primary and secondary endpoints are shown in Table 4. The CADILLAC risk score had the best discriminative accuracy, with an AUC of 0.8207 (p<0.0001), 0.8210 (p<0.0001), and 0.8192 (p<0.0001) for 6-, 12-, and 24-month mortality, respectively. Calibration of each risk score was performed by comparing predicted probabilities with 6-, 12-, and 24-month mortality estimates, and all models had an adequate goodness-of-fit (S1 Fig, S2 Fig, S3 Fig, and S4 Fig). The AUC calculated for each of risk score models for mortality at 24 months of follow up are shown in Fig 1 The highest performance of the CADILLAC risk score was observed. In addition, the CADILLAC risk score also had the best predictive value for in-hospital bleeding and acute renal failure, with an AUC of 0.7919 (p<0.05) and 0.9764 (p<0.0001), respectively. As for in-hospital repeated MI, the GRACE risk score had the highest predictive accuracy with an AUC of 0.9288 (p<0.05). Similarly, the GRACE score was the best predictive tool for new onset cardiogenic shock, with an AUC of 0.8648 (p<0.0001).

**Fig 2:**
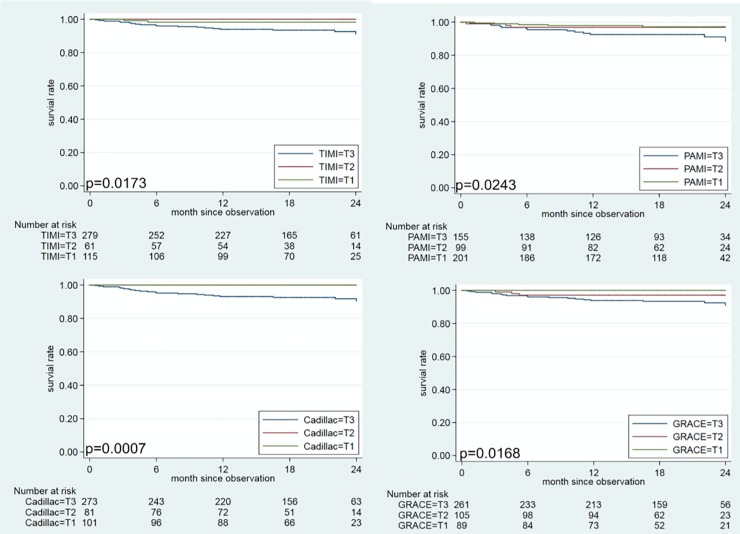
Kaplan-Meier overall survival curve for patients with STEMI stratified by tertiles of TIMI, PAMI, CADILLAC and GRACE risk scores. The cut-off points of these tertiles (T1-T3) are ≤2, 3, and ≥4 for TIMI; ≤3, 4–5, and ≥6 for PAMI; ≤3, 4–5, and ≥6 CADILLAC; ≤115, 116–137, and ≥138 for GRACE, respectively.

**Fig 3:**
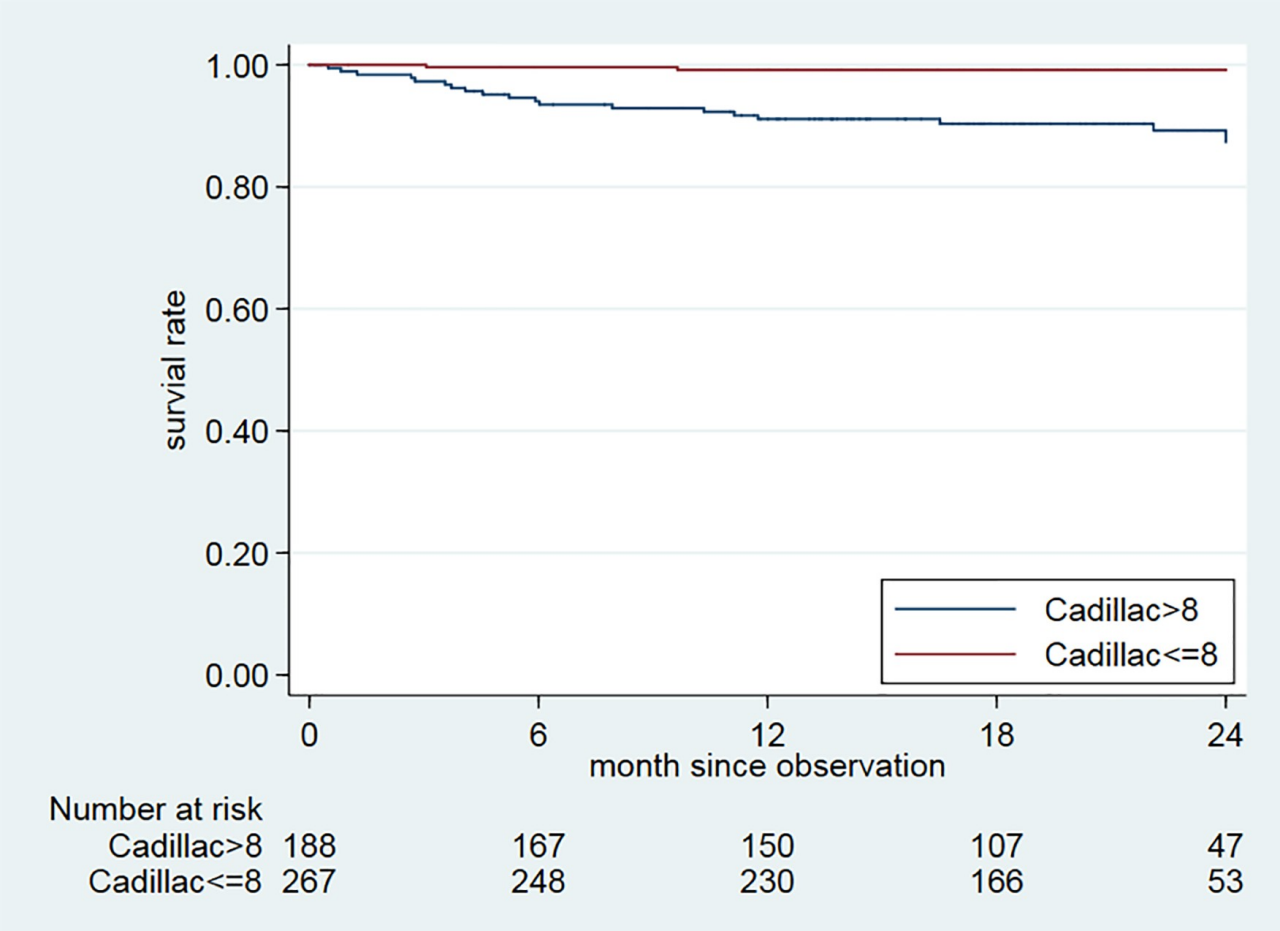
Observed survival by Controlled Abciximab and Device Investigation to Lower Late Angioplasty Complications (CADILLAC) scores. Two-year mortality of patients with CADILLAC scores ≤8 and >8 (for both, log-rank test p<0.0001).

**Table 4:** The AUC of four scoring models for primary and secondary endpoints.

Endpoint	TIMI		PAMI		CADILLAC		GRACE	
	AUC	95% CI	AUC	95% CI	AUC	95% CI	AUC	95% CI
**Primary endpoint—death**								
**6 months**	0.6942*^§^	0.5339–0.8546	0.6838*^§^	0.5045–0.8630	0.8207***	0.7294–0.9119	0.6661*^§^	0.5161–0.8161
**12 months**	0.7396**	0.6111–0.8681	0.7094**	0.5671–0.8518	0.8210***	0.7414–0.9006	0.7173*	0.5983–0.8362
**24 months**	0.7352**	0.6198–0.8506	0.7145***	0.5835–0.8455	0.8192***	0.7491–0.8894	0.7443**	0.6378–0.8508
**Secondary endpoints–in-hospital outcome**								
**Recurrent MI**	0.7788*	0.4699–1.0000	0.7434	0.3368–1.0000	0.7485	0.5167–0.9804	0.9288*	0.8738–0.9839
**Bleeding**	0.6158	0.3614–0.8702	0.6000^§^	0.3418–0.8583	0.7919*	0.6521–0.9318	0.5783^§^	0.3553–0.8013
**New onset cardiogenic shock**	0.7621*^§^	0.6382–0.8859	0.5771	0.4397–0.7145	0.6000	0.4529–0.7472	0.8648***^§^	0.7852–0.9444
**Acute renal failure**	0.8285^§§^	0.7587–0.8983	0.7131^§^	0.4585–0.9678	0.9764*	0.9498–1.0000	0.7795	0.5796–0.9794

**Abbreviations:** AUC, area under the receiver-operating characteristics curve; CADILLAC, Controlled Abciximab and Device Investigation to Lower Late Angioplasty Complications; CI, confidence interval; GRACE, Global Registry of Acute Coronary Events; MI, myocardial infarction; PAMI, Primary Angioplasty in Myocardial Infarction; TIMI, Thrombolysis In Myocardial Infarction.

*/**/*** Statistical significance of AUC p<0.05 /p<0.001 /p<0.0001

§/§§ Statistical significance of difference between AUC using DeLong’s test (reference model CADILLAC score) p<0.05/p<0.001.

**Table 5:** The AUC, sensitivity, specificity, Youden’s index, and cut-off point of each risk score.

	AUC	95% CI	Sensitivity	Specificity	Youden’s index	Cut-off point
**Death at 2 years**						
****TIMI	0.7352	0.6198–0.8506	0.5714	0.8318	0.4032	7.0005
****PAMI	0.7145	0.5835–0.8455	0.5714	0.7995	0.3710	6.9986
****CADILLAC	0.8192	0.7491–0.8894	0.9048	0.6106	0.5154	7.9989
****GRACE	0.7443	0.6378–0.8508	0.4129	0.9816	0.12448.	230.97
**Death at 1 year**						
****TIMI	0.7396	0.6111–0.8681	0.7222	0.7254	0.4476	6.0005
****PAMI	0.7094	0.5671–0.8518	0.4444	0.9130	0.3575	9.9983
****CADILLAC	0.8210	0.7414–0.9006	0.8889	0.6064	0.4953	8.0010
****GRACE	0.7173	0.5983–0.8362	0.2222	0.9291	0.1513	205.71
**Death at 6 months**						
****TIMI	0.6942	0.5339–0.8546	0.6923	0.7195	0.4118	6.0002
****PAMI	0.6838	0.5045–0.8630	0.4615	0.9095	0.3710	9.9990
****CADILLAC	0.8207	0.7294–0.9119	0.6923	0.7783	0.4706	10.9989
****GRACE	0.6661	0.5161–0.8161	0.2308	0.9276	0.1584	206.061

**Abbreviations:** AUC, area under the receiver-operating characteristics curve; CADILLAC, Controlled Abciximab and Device Investigation to Lower Late Angioplasty Complications; CI, confidence interval; GRACE, Global Registry of Acute Coronary Events; PAMI, Primary Angioplasty in Myocardial Infarction; Thrombolysis In Myocardial Infarction.

## Discussion

In the current study, 4 risk stratification models (TIMI, GRACE, PAMI, CADILLAC) were compared in Taiwanese diabetic patients diagnosed with STEMI according to the ACS guidelines of the TSOC. To our best knowledge, the present study is the first to demonstrate the relevant discriminatory ability of these four risk scores for mortality and clinical outcomes at time points up to two years in a cohort of diabetic patients with STEMI. The four models had good predictive value in estimating 2-year mortality, although the AUCs were slightly different. AUC values obtained from our database for predicting one-year mortality were even better than those described by the original authors [4,5]. Furthermore, the CADILLAC risk score had the best predictive value for bleeding and acute renal failure and the highest prognostic accuracy for mortality at each observed time point, including 6-month, 1-year, and 2-year. Our study demonstrated that the CADILLAC risk score is the best tool for prediction long-term mortality in Taiwanese diabetic patients diagnosed with STEMI, according to the nationwide real-world registry.

The predictive accuracy was 0.82 at 1-year follow-up for the CADILLAC risk score in our dataset, consistent with the fact that the score was originally developed to determine 1-year survival. In the present study, the CADILLAC risk score had a higher prognostic accuracy than the 0.74 1-year mortality prediction in the study by Kozieradzka et al [5]. Additionally, the AUC remained unchanged and retained a very good predictive power for 2-year mortality. It performed better than in the derivation and validation sets of the CADILLAC randomized clinical trial, in which the prognostic accuracy was 0.79 [4].

Some factors may explain the better prognostic power of the CADILLAC risk score in this study. First, the CADILLAC risk score was developed based on 1-year survival analysis. It is the only risk scoring model which takes into consideration ejection fraction and three-vessel disease. In a Korean clinical registry, LV dysfunction, poor TIMI flow after PCI, and multi-vessel disease were associated with long term major cardiovascular events after MI [17]. The presence of LV dysfunction assessed by baseline left ventriculography in patients who undergo PCI is a powerful predictor of early and late (3-year) mortality [18]. In this registry, 98% of the STEMI population received coronary angiogram. Detailed assessment of culprit lesions, TIMI flow and LV function were completed accordingly. These clinical data may provide additional prognostic relevant information for the study population. Those STEMI patients with LV dysfunction may develop cardiorenal syndrome, reflecting an abrupt worsening of cardiac function leading to acute kidney injury [19]. Second, elevated serum creatinine and anemia had been proved to be the independent baseline predictors to predict bleeding in patients with acute coronary syndrome [20]. The CADILLAC risk score is the only system that considers anemia and creatinine (renal insufficiency) in these 4 risk stratification models. Therefore, it should be more sensitive in predicting in-hospital bleeding and acute renal failure.

The GRACE score is based on a large registry of patients across the entire spectrum of coronary syndromes and is designed to determine all-cause mortality at 6 months [21,22]. The poor performance (0.67) of the GRACE score in predicting all-cause death at 6 months of our dataset was expected. The poor accuracy can be explained by the relatively lower number of Killip III/IV patients (around 20%). However, the GRACE score did have the highest prognostic accuracy for secondary endpoints regarding recurrent MI and new-onset cardiogenic shock (Table 4). The exact reason for this accuracy is not entirely clear. It may be due to the prior episode of cardiac arrest and the increased number of cardiac markers, both are the components of GRACE score, indicating myocardial damage in progress and causing further events such as cardiogenic shock and recurrent MI. The other 3 risk scoring models (TIMI, PAMI, and CADILLAC) do not include the component of cardiac arrest or increased cardiac markers. Therefore, GRACE score might be more sensitive in predicting in-hospital recurrent MI and new-onset cardiogenic shock.

Because of guideline-directed medical therapy and interventions, in-hospital and 1-year mortality rates for patients with STEMI have significantly decreased [23–27]. However, DM is still an independent predictor of 3-year mortality and 3-year major adverse cardiac events [28]. Therefore, it is important to use best practices guidelines to manage diabetic STEMI patients. Clinicians should also emphasize evidence-based medical therapies and available reperfusion therapy. For those diabetic STEMI patients with higher CADILLAC scores (>8), strict adherence to optimal medical treatment is mandatory.

Our work had three main limitations. Firstly, our dataset included 15% newly diagnosed DM patients. We could not record the exact diagnosis year of known diabetic patients in our cohort because of limited information. Nevertheless, there were no significant differences in adverse events between new-diagnosed and known diabetic patients [28]. Secondly, the study cohort was relatively small and limited in diabetic population. However, these patients were followed up prospectively and the data thoroughly analyzed. Finally, missing data prevented our application in our study cohort of newer risk scoring models such as the Syntax score. The angiography-based scoring model was created for predicting long-term major adverse cardiac events when treating severe coronary artery disease such as multi-vessel disease or left main coronary artery involvement. Fully 31.9% of patients of our dataset had three-vessel disease. Such patients should be evaluated for potential revascularization.

## Conclusions

Several risk scoring models showed a high predictive value to estimate 1-year mortality in Taiwanese diabetic STEMI patients. Among them, the CADILLAC system was superior at predicting 6-month, 1-year, and 2-year mortality. We should especially monitor patients with higher CADILLAC scores (>8). Strict adherence to medical therapy guidelines and intensive cardiovascular risk factor modification should be encouraged.

## Supporting information

S1 Fig Calibration plot for the TIMI score for 2-year, 1-year, and 6-month death. 2-year Hosmer and Lemeshow goodness-of-fit test p = 0.5400 1-year Hosmer and Lemeshow goodness-of-fit test p = 0.3991 6-month Hosmer and Lemeshow goodness-of-fit test p = 0.4618 **Abbreviation:** TIMI, Thrombolysis In Myocardial Infarction.
(DOCX)

S2 Fig Calibration plot for the PAMI score for 2-year, 1-year, and 6-month death. 2-year Hosmer and Lemeshow goodness-of-fit test p = 0.6786 1-year Hosmer and Lemeshow goodness-of-fit test p = 0.6422 6-month Hosmer and Lemeshow goodness-of-fit test p = 0.2774 **Abbreviation:** PAMI, Primary Angioplasty in Myocardial Infarction.
(DOCX)

S3 Fig Calibration plot for the CADILLAC score for 2-year, 1-year, and 6-month death. 2-year Hosmer and Lemeshow goodness-of-fit test p = 0.5416 1-year Hosmer and Lemeshow goodness-of-fit test p = 0.6505 6-month Hosmer and Lemeshow goodness-of-fit test p = 0.8121 **Abbreviation:** CADILLAC, Controlled Abciximab and Device Investigation to Lower Late Angioplasty Complications.
(DOCX)

S4 Fig Calibration plot for the GRACE score for 2-year, 1-year, and 6-month death. 2-year Hosmer and Lemeshow goodness-of-fit test p = 0.6369 1-year Hosmer and Lemeshow goodness-of-fit test p = 0.4677 6-month Hosmer and Lemeshow goodness-of-fit test p = 0.7567 **Abbreviation:** GRACE, Global Registry of Acute Coronary Events.
(DOCX)
