# Supplementary figures and images for: Comparison of the TIMI, GRACE, PAMI and CADILLAC risk scores for prediction of long-term cardiovascular outcomes in Taiwanese diabetic patients with ST-segment elevation myocardial infarction: From the registry of the Taiwan Society of Cardiology
Source: PLoS One. 2020 Feb 13;15(2):e0229186. doi: 10.1371/journal.pone.0229186 (PMC7018102; doi:10.1371/journal.pone.0229186)

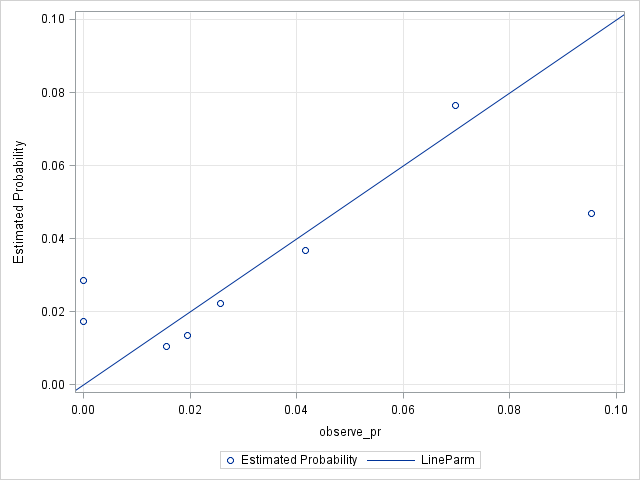

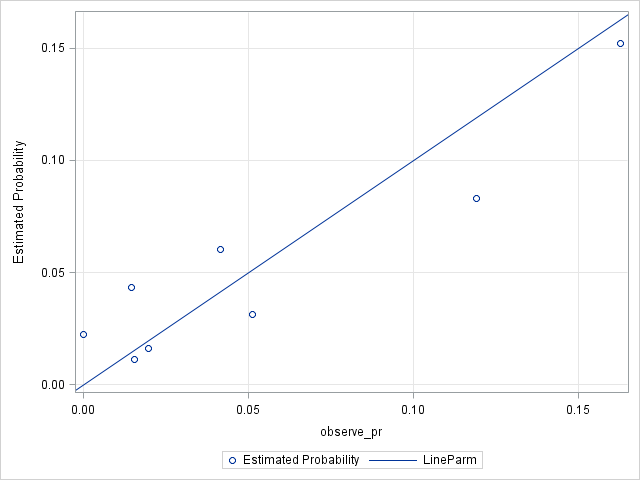

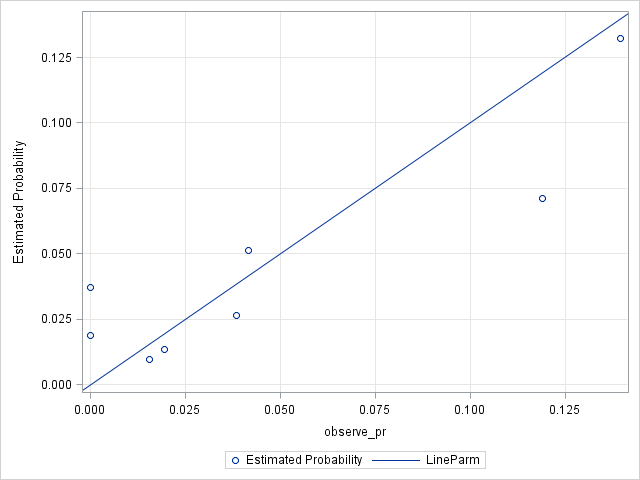
2-year 1-year 6-month

Supplement: S1 Fig — 2-year Hosmer and Lemeshow goodness-of-fit test p = 0.5400 1-year Hosmer and Lemeshow goodness-of-fit test p = 0.3991 6-month Hosmer and Lemeshow goodness-of-fit test p = 0.4618 Abbreviation: TIMI, Thrombolysis In Myocardial Infarction. (DOCX) [file pone.0229186.s001.docx]

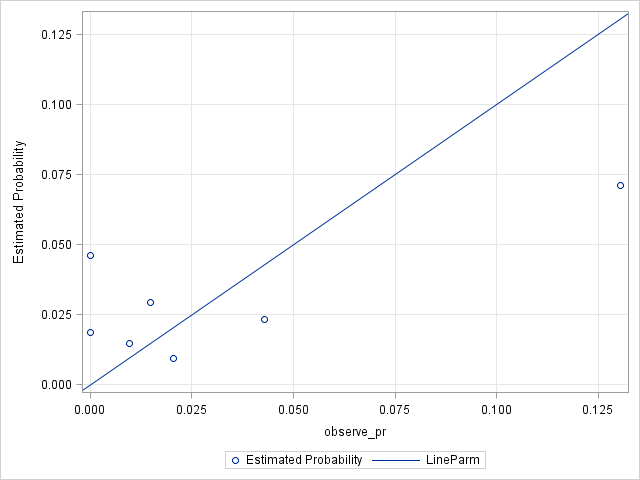

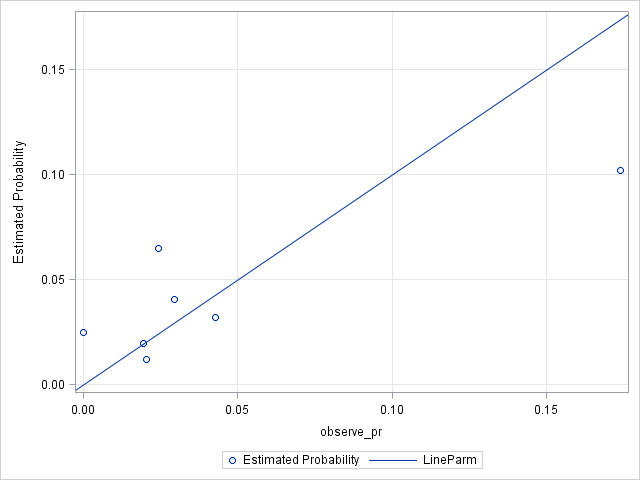

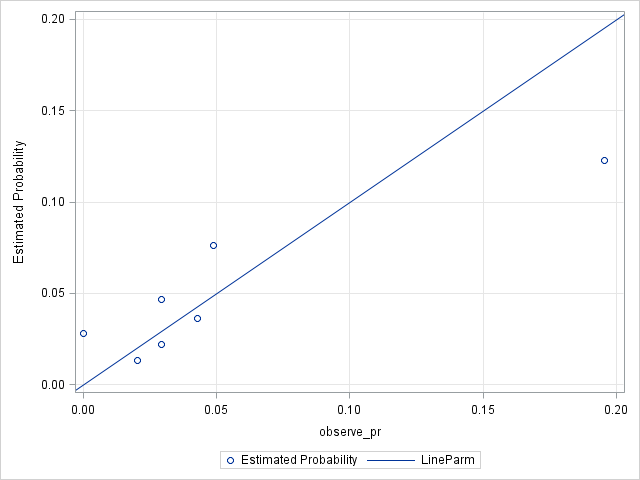
2-year 1-year 6-month

Supplement: S2 Fig — 2-year Hosmer and Lemeshow goodness-of-fit test p = 0.6786 1-year Hosmer and Lemeshow goodness-of-fit test p = 0.6422 6-month Hosmer and Lemeshow goodness-of-fit test p = 0.2774 Abbreviation: PAMI, Primary Angioplasty in Myocardial Infarction. (DOCX) [file pone.0229186.s002.docx]

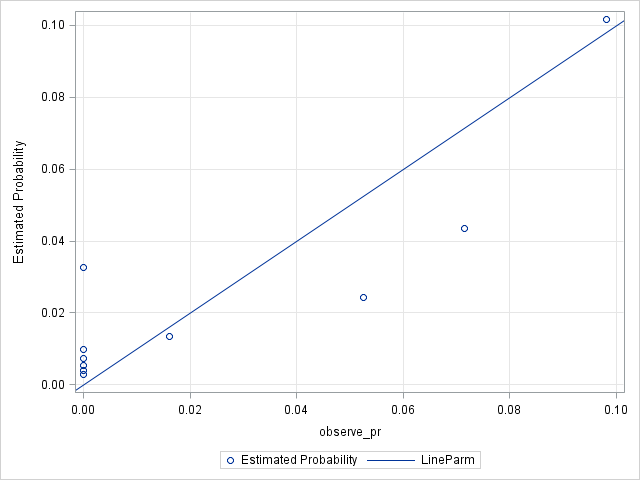

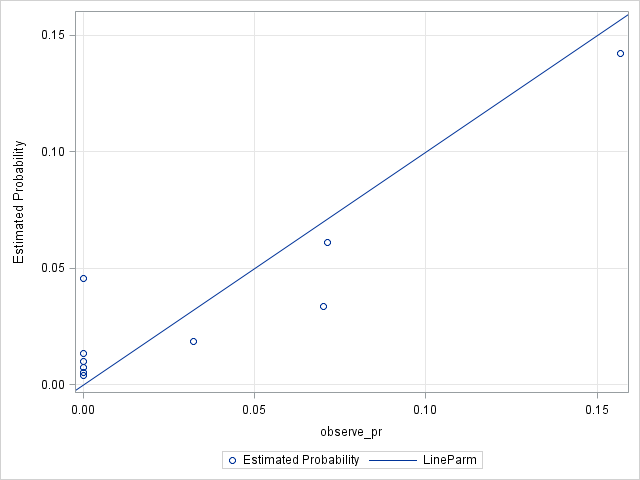

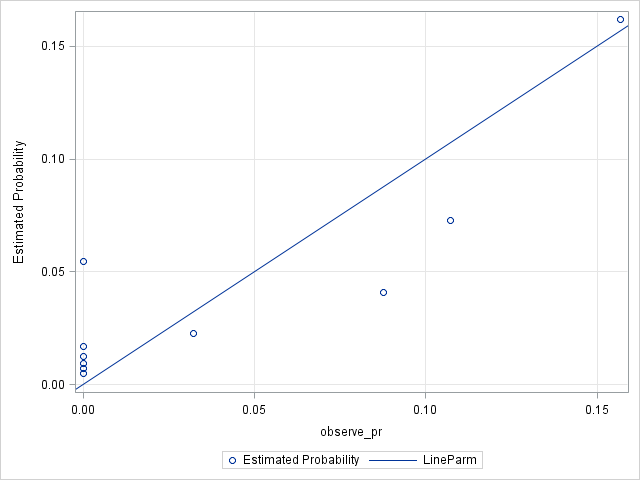
2-year 1-year 6-month

Supplement: S3 Fig — 2-year Hosmer and Lemeshow goodness-of-fit test p = 0.5416 1-year Hosmer and Lemeshow goodness-of-fit test p = 0.6505 6-month Hosmer and Lemeshow goodness-of-fit test p = 0.8121 Abbreviation: CADILLAC, Controlled Abciximab and Device Investigation to Lower Late Angioplasty Complications. (DOCX) [file pone.0229186.s003.docx]

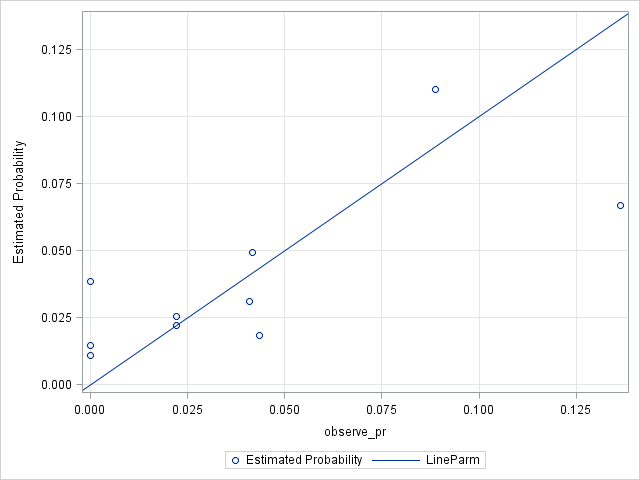

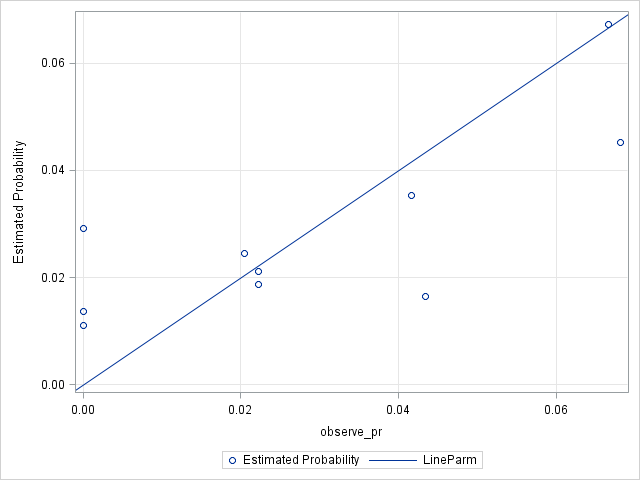

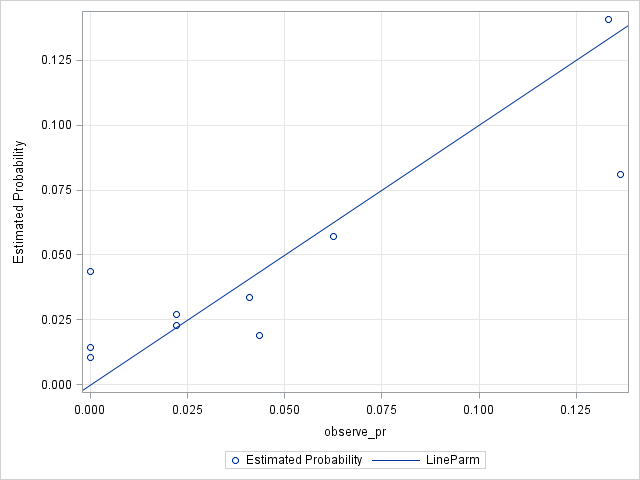
2-year 1-year 6-month

Supplement: S4 Fig — 2-year Hosmer and Lemeshow goodness-of-fit test p = 0.6369 1-year Hosmer and Lemeshow goodness-of-fit test p = 0.4677 6-month Hosmer and Lemeshow goodness-of-fit test p = 0.7567 Abbreviation: GRACE, Global Registry of Acute Coronary Events. (DOCX) [file pone.0229186.s004.docx]
